# ESCRT III-mediated lysosomal repair improve renal tubular cell injury in cisplatin-induced AKI

**DOI:** 10.1080/15548627.2025.2483598

**Published:** 2025-04-04

**Authors:** Zhangyu Tian, Yiming Wu, Bin Yi, Ling Li, Yan Liu, Hao Zhang, Aimei Li

**Affiliations:** a Department of Nephrology, The Third Xiangya Hospital, The Critical Kidney Disease Research Center, Central South University, Changsha, Hunan, China; b Center for Medical Genetics, School of Life Sciences, Central South University, Changsha, Hunan, China

**Keywords:** Apoptosis, autophagy, cisplatin-induced AKI, ESCRT III, lysosomal damage, lysosomal repair

## Abstract

The chemotherapeutic agent cisplatin is widely utilized for the treatment of various solid tumors. However, its clinical utility is limited by nephrotoxicity. Although numerous studies have demonstrated the potential of enhancing macroautophagy/autophagy in alleviating cisplatin-induced acute kidney injury (AKI), the dynamics of the autophagic process during renal tubular injury remain to be elucidated. In our investigation, we observed that cisplatin treatment leads to increased expression of LC3-II, GABARAPL1, SQSTM1/p62 and NBR1 in mouse renal tubular epithelial cells and BUMPT cells. Moreover, ultrastructurally, there is extensive accumulation of autophagic vacuoles in AKI mice. These findings imply that cisplatin-induced AKI results in impaired autophagic flow within renal tubular cells. Furthermore, LGALS3 (galectin 3) was found to be enriched in lysosomes after cisplatin treatment, revealing a close association between autophagy dysfunction and impaired lysosomal membrane integrity. Given the damaging contents of lysosomes, lysosomal membrane permeabilization must be rapidly resolved. Our findings showed that ESCRT III subunit CHMP4A-mediated lysosomal membrane repair significantly ameliorates autophagic defects and protects against lysosomal damage-induced cell death in a cisplatin-induced AKI model. In conclusion, our study indicates that ESCRT III-mediated lysosomal repair can relieve cisplatin-induced cell apoptosis and restore normal autophagy function in renal tubular epithelial cells. This mechanism plays a protective role against cisplatin-induced AKI.

**Abbreviations:** AAV: adeno-associated virus; AKI: acute kidney injury; CQ: chloroquine; ESCRT: endosomal sorting complex required for transport; LMP: lysosomal membrane permeabilization; MAP1LC3/LC3: microtubule associated protein 1 light chain 3; MTOR: mechanistic target of rapamycin kinase; PAS: periodic acid Schiff; PTECs: proximal renal tubule epithelial cells; TEM: transmission electron microscopy; TUNEL: terminal deoxynucleotidyl transferase dUTP nick end labeling.

## Introduction

Acute kidney injury (AKI) constitutes a major complication among hospitalized patients, with a high global mortality burden at 20%~50% [1,2]. Cisplatin plays a crucial role in treating various solid tumors. However, its clinical utility is limited due to the development of AKI in approximately 30% of cisplatin-treated patients [3]. Cisplatin enters the cell through receptors on proximal tubular epithelial cells and gradually accumulates, where it can cross-link with bases in DNA, triggering DNA damage, oxidative stress, apoptosis, and macroautophagy/autophagy [4–6]. Current strategies for preventing or treating cisplatin-induced AKI are limited, necessitating further exploration of precise molecular mechanisms.

Lysosomes serve as pivotal catabolic organelles with the functionality contingent upon the acidic lumenal pH and diverse hydrolytic enzymes within the lysosomal lumen [7,8]. Lysosomes serving as the final degradation sites for substances transported via endocytosis and autophagy pathways are notably susceptible to injury [9]. Lysosomes are key regulators of cellular metabolism, influencing both catabolic and anabolic processes through direct interactions with MTOR (mechanistic target of rapamycin kinase) and AMP-activated protein kinase (AMPK) [10]. Studies have suggested that improved lysosomal functions and enhanced autophagic flux could protect against renal tubular cell apoptosis and kidney injury induced by cisplatin, which may via Activation of TFEB [11]. Cisplatin-induced AKI initiates DNA damage and activates ROS, apoptotic factors (BCL-family, caspases) and inflammatory cytokines [3,12], potentially resulting in lysosomal membrane permeabilization (LMP), which poses a significant risk to cellular homeostasis and may trigger cell death [13,14]. LMP leads to the release of cathepsins and other hydrolases from the lysosomal lumen into the cytosol, crucial for initiating apoptosis [15,16]. The most abundant lysosomal proteases are CTSB (cathepsin B) and CTSD, followed by CTSL [17,18]. CTSB plays a crucial role in the processing of BID and CASP2 (caspase 2) [19], as well as in the production of reactive oxygen species [20]. Alongside other cathepsins, CTSB contributes to the degradation of BCL2, MCL1, and BCL2L11/BimEL [21,22]. These processes ultimately lead to mitochondrial membrane permeabilization and the release of cytochrome c [22]. In AKI, damaged lysosomes fail to degrade impaired organelles and cells worsening the condition. CTSB and CTSL are increased in renal ischemia/reperfusion injury, and hydroxychloroquine attenuates renal injury by downregulating CTSB- and CTSL-mediated activation of the NLRP3 inflammasome [16]. CTSB was found to contribute to the pathogenesis of sepsis-induced acute kidney injury, and the CTSB inhibitor CA074 was shown to alleviate this condition [23]. Hence, mitigating lysosomal damage and maintaining lysosomal integrity are essential for maintaining lysosomal function to ameliorate cell death.

Lysosomal injury triggers the endo-lysosomal damage response (ELDR), encompassing three branches – rapid lysosomal repair mediated by the endosomal sorting complex required for transport (ESCRT) and phosphoinositide-initiated membrane tethering and lipid transport (PITT) pathways for mild injury, activated lysophagy and microautophagy for irreparable damage, and TFEB- and TFE3-driven lysosomal biogenesis [24]. Upon lysosomal damage, recruits and organizes ESCRT components such as PDCD6IP/ALIX, CHMP4A, and CHMPB at the damaged sites, facilitating ESCRT-driven lysosomal membrane repair [10]. Certain galectins, long recognized as markers of lysosomal membrane damage, accumulate on damaged lysosomes [25]. Among them, LGALS3 (galectin 3) plays a crucial role in lysosomal repair by dynamically associating with ESCRT components, thereby recruiting ESCRT III proteins to facilitate membrane repair [10]. The relationship of lysosome damage and cisplatin-induced AKI, and the role of ESCRT III-mediated lysosomal repair are unclear.

In this study, we investigated the role of lysosomal repair in cisplatin-induced AKI, and the mechanisms of this effect. Our results show that cisplatin treatments induce autophagy dysfunction and lysosomal damage, which further cause cell death in renal tubular epithelial cells. Furthermore, knockdown of ESCRT III exacerbates cisplatin-induced apoptosis and disrupted autophagy. However, overexpression of ESCRT III can repair cisplatin-induced lysosomal membrane permeabilization, ameliorate autophagic dysfunction and protects against lysosomal damage-induced cell death. Our study demonstrates that ESCRT III-mediated lysosomal repair plays a pivotal role in AKI and may be a potential therapeutic target for cisplatin nephrotoxicity.

## Results

### Cisplatin-induced AKI increased apoptosis and autophagic disorders in proximal tubular epithelial cells

**Figure 1.**
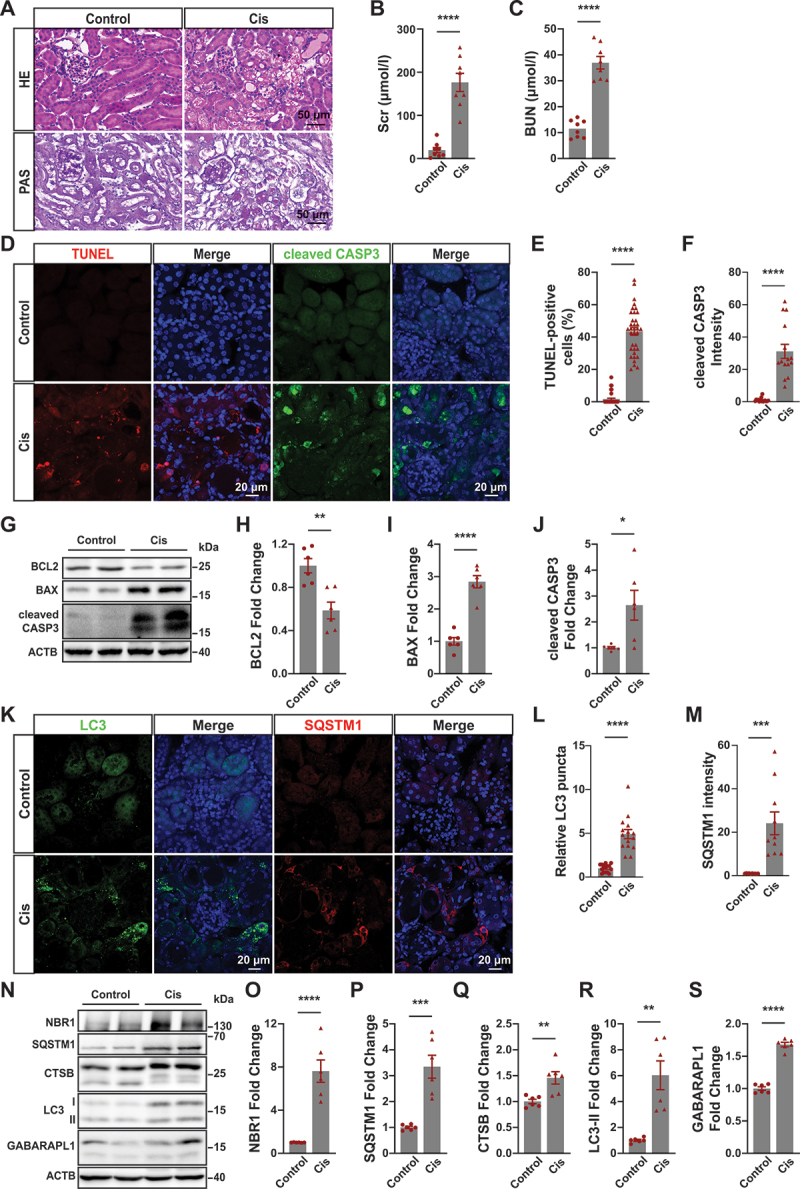
Cisplatin-induced AKI increased apoptosis and autophagic disorders in proximal tubular epithelial cells of mice. (A) HE and PAS staining of kidney sections of mice treated with normal saline or cisplatin (cis, 25 mg/kg body weight). Scale bar: 50 μm. (B) Serum creatinine of mice in different treatment groups as indicated. ****p < 0.0001 vs control group. *n* = 8. (C) Blood Urea Nitrogen (BUN) of mice in different treatment groups as indicated. ****p < 0.0001 vs control group. *n* = 8. (D) TUNEL staining and immunofluorescence staining of cleaved CASP3 in proximal tubule epithelial cells from mice of each group as indicated. Scale bar: 20 μm. (E-F) Tunel-positive cells and cleaved CASP3-positive cells were quantified from proximal tubular epithelial cells of mice in each group. ****p < 0.0001 vs control group. *n* = 8. (G) Western blot analysis of BCL2, BAX and cleaved CASP3 in the renal cortex of mice from each group as indicated. ACTB/β-actin was used as the loading control. (H-J) Quantitative analysis of the data in (G) by ImageJ software. *p < 0.05, **p < 0.01, ****p < 0.0001 vs control group. *n* = 8. (K) immunofluorescence analysis of LC3 and SQSTM1 in proximal tubule epithelial cells from mice of each group as indicated. Scale bar: 20 μm. (L-M) Quantitative analysis of the data in (K), ***p < 0.001, ****p < 0.0001 vs control group. *n* = 8. N: Western blot analysis of NBR1, SQSTM1, CTSB, LC3 and GABARAPL1 in the renal cortex of mice from each group as indicated. ACTB was used as the loading control. (O-S) Quantitative analysis of the data in (N) by ImageJ software. **p < 0.01, ***p < 0.001, ****p < 0.0001 vs control group. *n* = 8.

Seventy-two hours after cisplatin administration, histological examination of renal tissue samples from mice revealed varying degrees of damage to the proximal renal tubule epithelial cells (PTECs) using HE and PAS staining techniques (Figure 1A). The levels of serum creatinine (Scr) and blood urea nitrogen (BUN) in the cisplatin-treated group were significantly elevated when compared to the control group, confirming successful construction of the AKI model (Figure 1B,C). TUNEL staining showed cisplatin induced PTECs apoptosis in mice, and immunofluorescence showed increased cleaved CASP3 expression in the cisplatin-treated group (Figure 1D–F). The levels of cleaved CASP3 and BAX were higher and BCL2 was lower in the cisplatin-treated group than that in the control group (Figure 1G–J). These results suggest that cisplatin increases apoptosis in proximal tubular epithelial cells of mice.

We investigated MAP1LC3/LC3 as a key marker for autophagy activation by examining its presence within the renal cortex. As depicted in Figure 1K, there was an increase in LC3 puncta formation among mice treated with cisplatin. To determine whether this increase indicated autophagic activation or impaired degradation processes, we assessed SQSTM1/p62 as an autophagy substrate. Notably, higher expression levels of SQSTM1 were detected in AKI mice induced by cisplatin when compared to wild-type mice, thus implying defective autophagy within these animals (Figure 1K–M). Immunoblot analysis further confirmed upregulation of Atg8 orthologs such as LC3-II and GABARAPL1 alongside increased expression levels of autophagy receptors including SQSTM1 and NBR1 (Figure 1N–S). The significant accumulation of these autophagic substrates suggests impairment of autophagic flux. Overall, these findings confirm that cisplatin induces autophagic flux impairment in AKI mice.

**Figure 2.**
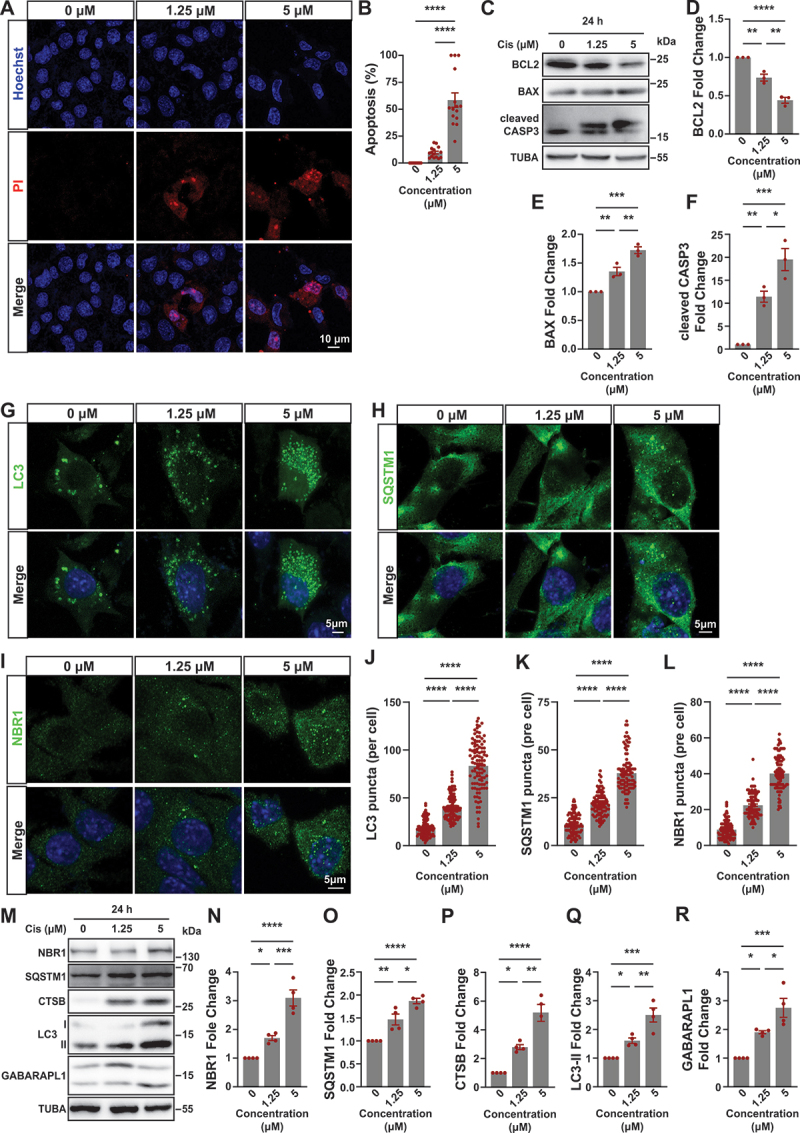
Cisplatin induced apoptosis and autophagy disorder in BUMPT cells. (A) PI and Hoechst double staining of BUMPT cells in each group as indicated. Scale bar: 10 μm. (B) Percentages of apoptosis in (A) were quantified. ****p < 0.0001. (C) Western blot analysis of BCL2, BAX and cleaved CASP3 of BUMPT cells in each group as indicated. TUBA/α-tubulin was used as the loading control. (D-F) Quantitative analysis of the data in (C) by ImageJ software. *p < 0.05, **p < 0.01, ****p < 0.0001. (G) Immunofluorescence analysis of LC3 puncta in BUMPT cells after intervention of cisplatin with different concentrations. Scale bar: 5 μm. (H) Immunofluorescence analysis of SQSTM1 puncta in BUMPT cells after intervention of cisplatin with different concentrations. Scale bar: 5 μm. (I) Immunofluorescence analysis of NBR1 puncta in BUMPT cells after intervention of cisplatin with different concentrations. Scale bar: 5 μm. (J-L) Quantitative analysis of the data in (G), (H), (I). ****p < 0.0001. (M) Western blot analysis of NBR1, SQSTM1, CTSB, LC3 and GABARAPL1 in BUMPT cells after intervention of cisplatin with different concentrations. TUBA was used as the loading control. (N-R) Quantitative analysis of the data in (M) by ImageJ software. Cell quantification consists of 100 cells in three independent experiments. *p < 0.05, **p < 0.01 vs control group.

Next, we explored the effect of cisplatin on apoptosis and autophagy in vitro. Then, we used mouse proximal tubular (BUMPT) cells to induce acute kidney injury. Cisplatin at 0, 1.25, 2.5, 5, 10 and 20 μM concentrations were used to determine the optimal concentration. Cisplatin treatment led to cell morphologies change which was noticeable at 20 μM, many cells showed cellular shrinkage and blebbing (Figure S1A). For the exploration of the influence of cisplatin in proliferation of BUMPT cells, CCK‐8 assays were used to detect the viability of cell after treatment with various concentrations of cisplatin for 24 h. As presented in Figure S1B, cisplatin inhibited the growth of BUMPT cells in a dose‐dependent manner. Based on these results, subsequent experiments were conducted using concentrations of 1.25 μM and 5 μM of cisplatin. PI and Hoechst double staining revealed a small number of apoptotic cells at a concentration of 1.25 μM, while significant apoptosis was observed following treatment with 5 μM cisplatin (Figure 2A,B). Immunoblot analysis demonstrated that at a concentration of 1.25 μM, cisplatin induced increased expression levels of BAX and cleaved CASP3, along with decreased expression levels of BCL2; these effects were more pronounced at a concentration of 5 μM (Figure 2C–F). Collectively, these findings indicate that cisplatin induce apoptosis in BUMPT cells.

In BUMPT cells, immunofluorescence staining showed an increase in the number of LC3 puncta and expression levels of SQSTM1 and NBR1 following treatment with 1.25 μM cisplatin, which became more pronounced after treatment with 5 μM cisplatin (Figure 2G–L). In addition, western blot showed a dose-dependent increase in the expression of NBR1, SQSTM1, CTSB, LC3-II, and GABARAPL1 upon cisplatin treatment (Figure 2M–R), which consistent with the in vivo results (Figure 1K–S). These results further support the notion that cisplatin induces dysfunction in autophagy within proximal tubular epithelial cells.

### Cisplatin induced lysosomal damage in proximal tubular epithelial cells

**Figure 3.**
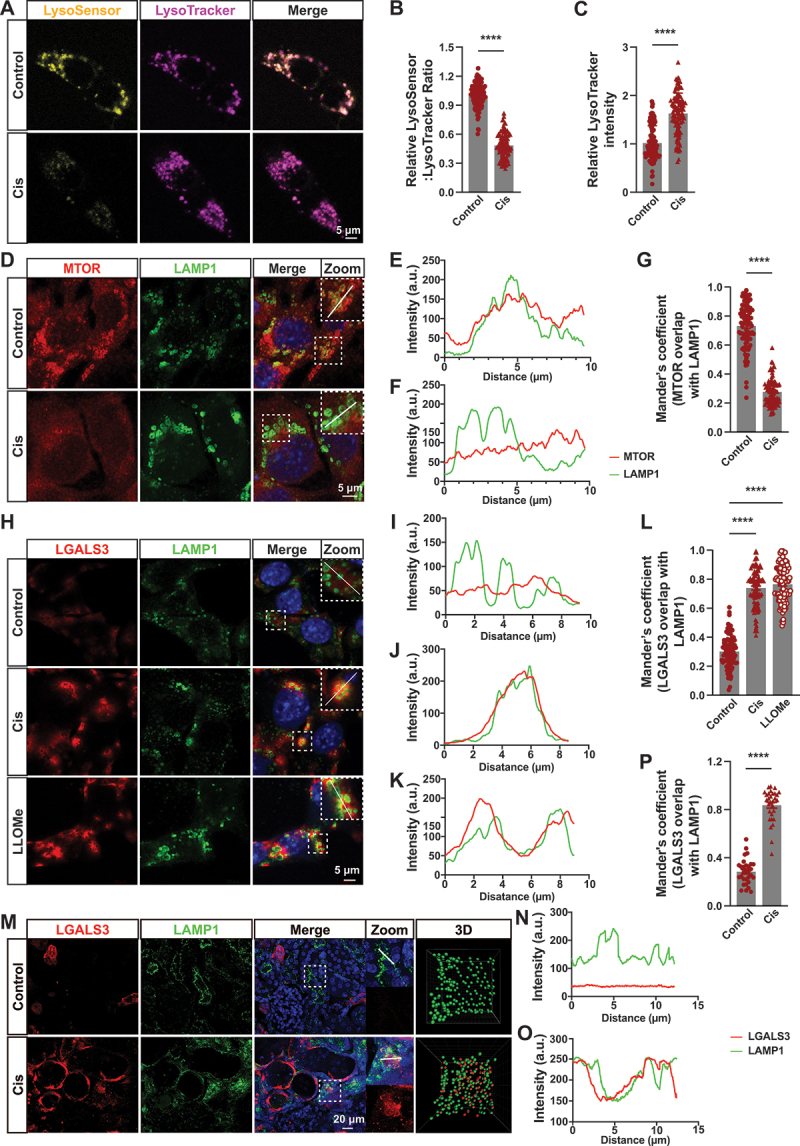
Cisplatin induced lysosome damage in proximal tubular epithelial cells. (A) LysoSensor and LysoTracker staining of BUMPT cells of each group as indicated. Scale bar: 5 μm. (B) Relative LysoSensor:LysoTracker intensity of the data in (A) was quantified. ****p < 0.0001 vs control group. (C) Relative LysoTracker intensity of the data in (A) was quantified. Cell quantification consists of 100 cells in three independent experiments. ****p < 0.0001 vs control group. (D) Immunofluorescence confocal microscopy of MTORC1 localization relative to LAMP1-positive lysosomes in BUMPT cells of each group as indicated. Scale bar: 5 μm. (E) Intensity within the indicated white area in control group was measured. A.U., arbitrary units. (F) Intensity within the indicated white area in cisplatin group was measured. (G) Manders’ overlap coefficients were determined. Cell quantification consists of 100 cells in three independent experiments. ****p < 0.0001 vs control group. (H) Immunofluorescence confocal microscopy of LGALS3 localization relative to LAMP1-positive lysosomes in BUMPT cells of each group as indicated. Scale bar: 5 μm. (I) Intensity within the indicated white area in control group was measured. (J) Intensity within the indicated white area in cisplatin group was measured. (K) Intensity within the indicated white area in LLOMe group was measured. (L) Manders’ overlap coefficients were determined. Cell quantification consists of 100 cells in three independent experiments. ****p < 0.0001 vs control group. (M) Immunofluorescence confocal microscopy of LGALS3 localization relative to LAMP1-positive lysosomes in PTECs from mice of each group as indicated. Scale bar: 20 μm. The 3D model visualization was performed using Imaris software. (N) Intensity within the indicated white area in control group was measured. (O) Intensity within the indicated white area in cisplatin group was measured. (P) Manders’ overlap coefficients were determined. ****p < 0.0001 vs control group. *n* = 8.

As cisplatin induced autophagic disorder and increased the expression of CTSB (lysosomal protease, Figures 1N and 2M), we employed two highly sensitive lysosomotropic probes to assess the impact of cisplatin on lysosomal function in renal tubular cells. LysoSensor staining, a widely utilized technique for measuring and quantifying lysosomal pH, demonstrated an increase in fluorescence intensity that was dependent on the acidification process. As shown in Figure 3A, the granular yellow fluorescence intensity exhibited a decrease following treatment with cisplatin, indicating an elevation in lysosomal pH. In the meantime, LysoTracker is mainly used for lysosomal distribution and labeling. Both LysoSensor and LysoTracker can be used in combination, with LysoSensor reflecting changes in the acidic environment and LysoTracker indicating lysosomal presence. Cisplatin attenuated lysosomal acidification (as evidenced by decreased ratio of LysoSensor:LysoTracker intensity, Figure 3B) and induced damaged lysosomal accumulation (Figures 3C, 1N, and 2M) in comparison to control. Collectively, these data provide additional validation for cisplatin-induced alkalinization of lysosomes in renal tubular cells. Studies had proved that lysosomal damage could inhibit MTOR activity [26] and active TFEB [27]. Under normal conditions, MTORC1 locates at lysosomal membrane. However, upon cisplatin treatment, MTORC1 translocated from lysosomes to the cytosol, the colocalization of MTORC1 and LAMP1 was diminished in BUMPT cells (Figure 3D–G). In the same time, we observed diminished MTOR activity as detected by phosphorylation of its substrates EIF4EBP1 and RPS6KB1 (Figure S2A-C). Furthermore, TFEB is localized in the cytoplasm under normal conditions. However, in response to treatment with either cisplatin or Torin1 (MTOR inhibitor), was observed to translocate to the nucleus (Figure S2D and E). Our data demonstrated that cisplatin inhibits MTOR activity and facilitates TFEB nuclear translocation, which further suggests lysosomal dysfunction induced by cisplatin.

To investigate this possibility, we employed LGALS3 as a marker for detecting damaged endomembrane [28]. When endosomal membrane ruptured, LGALS3 could access the luminal glycoproteins of these compartments. Under untreated conditions, LGALS3 was diffusely distributed throughout the cytosol and did not colocalize with LAMP1, a lysosomal membrane protein. However, upon cisplatin or LLOMe (l-leucyl-l-leucine methyl ester, an inducer of lysosomal membrane permeabilization) treatment, several LGALS3 puncta appeared in the cytosol and extensively colocalized with LAMP1 in PETCs of mice and BUMPT cells (Figure 3H–O). The same results were further corroborated in mice: We observed that LGALS3 aggregated into punctate structures, which colocalized with lysosomes in the proximal tubular epithelial cells after AKI (Figure 3O,P). Additionally, we used Imaris software to construct 3D models for clearer visualization of the colocalization between LAMP1 and LGALS3 (Figure 3M). These findings strongly suggest that cisplatin possesses the capability to disrupt lysosomal membrane in proximal tubular epithelial cells.

### Cisplatin inhibits the formation of autolysosomes by impairing lysosomes in proximal tubular epithelial cells

**Figure 4.**
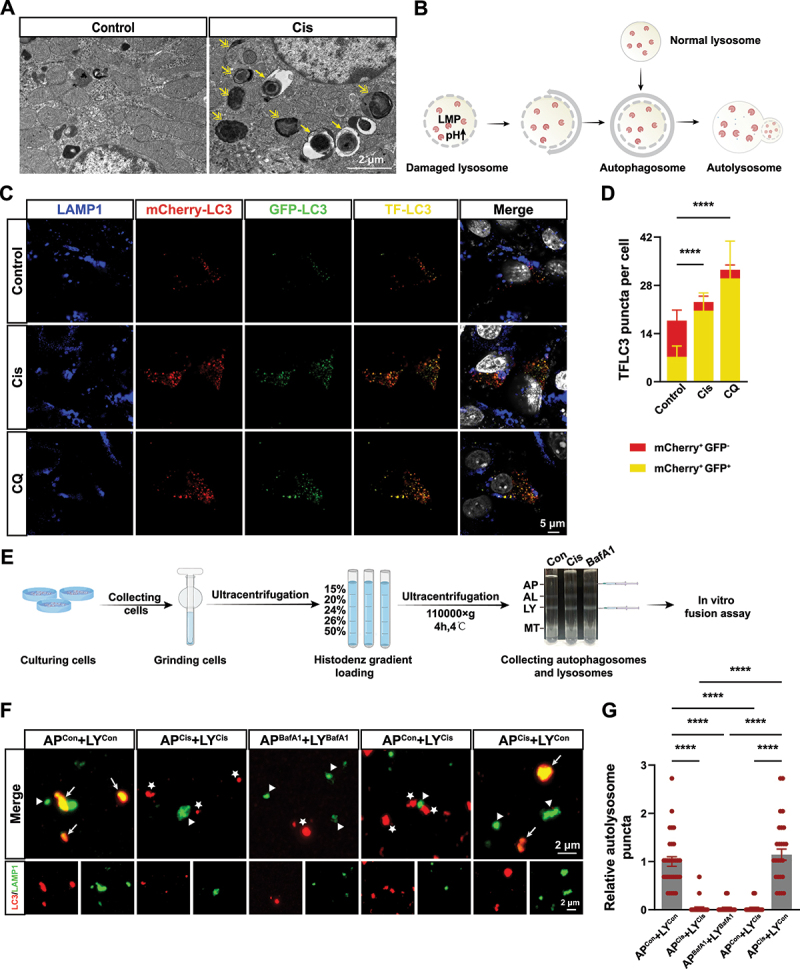
Cisplatin inhibits the formation of autolysosomes by impairing lysosomes in BUMPT cells. (A) TEM of proximal tubule epithelial cells from mice of each group as indicated. The arrows indicate autophagosome. The double arrows indicate autolysosome. Scale bar: 2 μm. (B) Schematic representation of autophagy triggered by damaged lysosomes. (C) BUMPT cells were transfected with TF-LC3, and then treated with control, cisplatin or CQ. The autophagosomes were shown as yellow puncta with both mCherry (red) and GFP (green) labels, autolysosome was shown as red only puncta because after fusion with lysosomes, GFP loses its fluorescence in acidic pH. Scale bar: 5 μm. (D) Quantitative analysis of the red and yellow puncta in (C). Cell quantification consists of 100 cells in three independent experiments. ****p < 0.0001 vs control group. (E) Simplified flowchart of the in vitro fusion experiment. BafA1, bafilomycin A_1_; AP, autophagosome; AL, autolysosome; LY, lysosome; MT, mitochondrion. (F) Immunofluorescence confocal microscopy of fusion of AP and LY in each group. The arrows indicate autolysosomes; the triangles indicate lysosome; the pentagrams indicate autophagosome. Scale bar: 5 μm. (G) Quantitative analysis of the data in (F). ****p < 0.0001.

Using transmission electron microscopy to examine ultrastructural changes in mouse renal tubules, following cisplatin treatment, we observed an increase in autophagy vacuoles within PTECs (Figure 4A), including autophagosomes (indicated by single arrows) and autolysosomes (indicated by double arrows), as shown in the schematic diagram in Figure 4B.

Furthermore, we used TF-LC3 to investigate the autolysosome maturation process. As mCherry is more stable than GFP in the acidic environment of lysosome, the normal maturation of autolysosomes is characterized with increased red-only puncta. In contrast, colocalization of GFP and mCherry puncta would indicate disruption of the fusion between autophagosomes, which presented with yellow puncta, with lysosomes [29]. Our results show that cisplatin treatment can activate autophagy leading to an increase in autophagic vacuoles, including both autolysosomes (red dots) and autophagosomes (yellow dots) (Figure 4C,D). Additionally, compared to the control group, cisplatin treatment resulted in altered lysosomal acidity and impaired autolysosome maturation, consistent with the chloroquine (CQ) group.

To further investigate why lysosomal damage results in impaired autolysosome maturation, we conducted in vitro fusion experiments (Figure 4E). The results of these experiments show that, compared to the control group cisplatin treatment impairs the fusion of autophagosomes with lysosomes which is consistent with the results from the bafilomycin A_1_ (BafA1) treatment group (Figure 4F,G). The abnormal lysosomes in the cisplatin group do not fuse with autophagosomes from the control group. However, autophagosomes from the cisplatin group are able to fuse with lysosomes from the control group (Figure 4F,G). This finding suggests that cisplatin treatment leads to impaired autophagosome-lysosome fusion due to lysosomal dysfunction.

### Lysosomal damage plays a significant role in cisplatin-induced apoptosis

**Figure 5.**
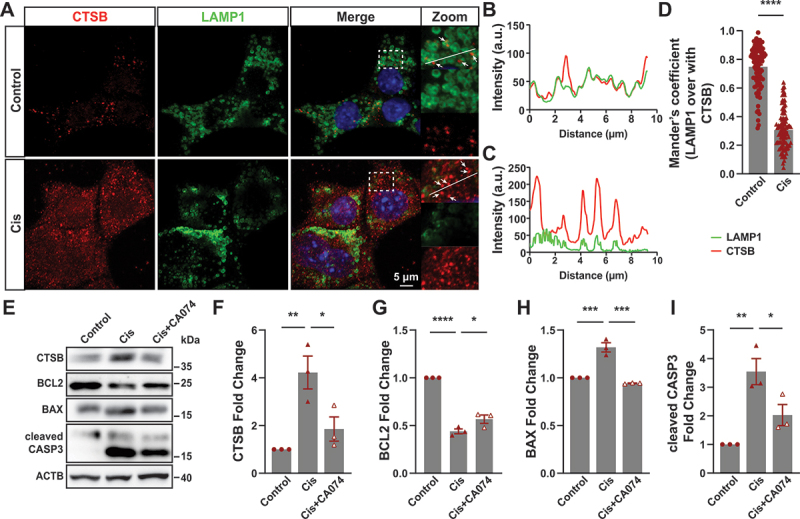
Lysosomal damage was involved in cisplatin-induced apoptosis in BUMPT cells. (A) Immunofluorescence confocal microscopy of CTSB localization relative to LAMP1-positive lysosomes in BUMPT cells of each group as indicated. The arrows indicate CTSB puncta. Scale bar: 5 μm. (B) Intensity within the indicated white area in control group was measured. (C) Intensity within the indicated white area in cisplatin group was measured. (D) Manders’ overlap coefficients were determined. Cell quantification consists of 100 cells in three independent experiments. ****p < 0.0001 vs control group. (E) Western blot analysis of CTSB, BCL2, BAX and cleaved CASP3 of BUMPT cells in each group as indicated. ACTB was used as the loading control. (F-I) Quantitative analysis of the data in (E) by ImageJ software. *p < 0.05, **p < 0.01, ***p < 0.001, ****p < 0.0001.

Studies had reported that lysosomal membrane permeabilization is associated with cell death, which led to increased cytoplasmic CTSB and CTSD [30]. Cathepsins release from the lysosomal lumen could activate apoptotic effectors to trigger apoptotic cell death [31]. Our results found that under normal conditions, CTSB was localized in lysosomes. However, upon treatment with cisplatin, CTSB exhibited a diffuse distribution throughout the cytoplasm, indicating its release from lysosomes into the cytosol (Figure 5A–D). To investigate whether the release of CTSB from lysosome to the cytosol was involved in cisplatin induced apoptosis in BUMPT cells, cells were pre-incubated with CA074 (CTSB inhibitor) following 24-h cisplatin treatment. Results showed that inhibition of cathepsins significantly prevented cisplatin-induced apoptosis (Figure 5E–I). Collectively, these findings strongly support the hypothesis that LMP-mediated release of lysosomal enzymes plays a crucial role in cisplatin-induced apoptosis in BUMPT cells.

### ESCRT III is required for improving cisplatin induced lysosomal repair and autophagy disorder in BUMPT cells

**Figure 6.**
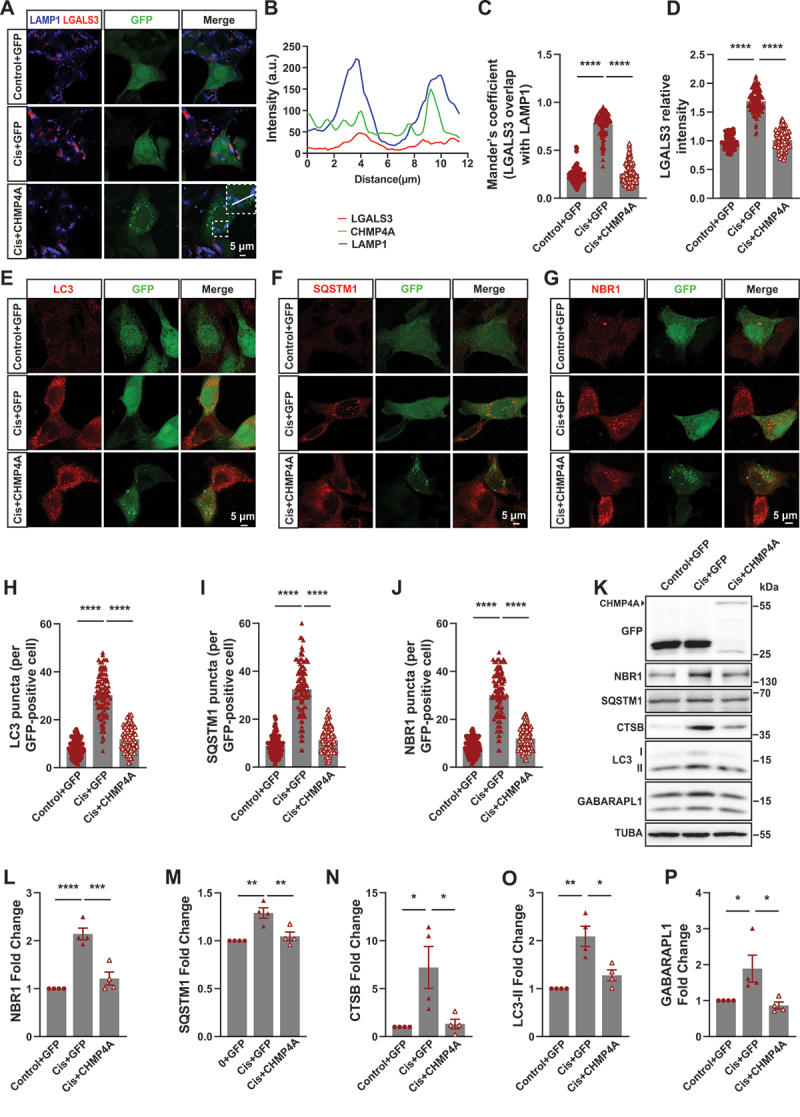
ESCRT III repaired lysosome damage and improved autophagy disorder induced by cisplatin. (A) Immunofluorescence staining of LGALS3 localization relative to LAMP1-positive lysosomes in BUMPT cells treated with DMSO or cisplatin after pEGFP-C1 or GFP-CHMP4A overexpression is shown. Scale bar: 5 μm. (B) Intensity within the indicated white area in cisplatin+CHMP4A group was measured. (C) Manders’ overlap coefficients were determined. Cell quantification consists of 100 cells in three independent experiments. ****p < 0.0001. (D) Intensity of LGALS3 in each group of (A) were quantified. (E) Immunofluorescence confocal microscopy of LC3 in BUMPT cells transfected with pEGFP-C1 or GFP-CHMP4A, and then treated with DMSO or cisplatin. Scale bar: 5 μm. (F) Immunofluorescence confocal microscopy of SQSTM1 in BUMPT cells transfected with pEGFP-C1 or GFP-CHMP4A, and then treated with DMSO or cisplatin. Scale bar: 5 μm. (G) Immunofluorescence confocal microscopy of NBR1 in BUMPT cells transfected with pEGFP-C1 or GFP-CHMP4A, and then treated with DMSO or cisplatin. Scale bar: 5 μm. (H-J) quantitative analysis of the data in (E-G). ****p < 0.0001. (K) Western blot analysis of GFP, NBR1, SQSTM1, CTSB, LC3 and GABARAPL1 of BUMPT cells transfected with pEGFP-C1 or GFP-CHMP4A, and then treated with DMSO or cisplatin. TUBA was used as the loading control. (L-P) quantitative analysis of the data in (K) by ImageJ software. *p < 0.05, **p < 0.01, ***p < 0.001, ****p < 0.0001.

Current studies have shown that lysosomal repair can alleviate autophagic dysfunction caused by lysosomal membrane damage and restore cellular physiological function to normal state. The ESCRT family consists of four complexes: ESCRT 0, I, II, and III [32]. ESCRT 0 is responsible for recruitment and initiation, ESCRT I and II mediate membrane budding, and ESCRT III is primarily responsible for membrane scission (Figure S3A). Among these, ESCRT III plays the most critical role in membrane repair [33]. ESCRT III is a dynamic complex (Figure S3B), composed of four core subunits (CHMP4, CHMP2, CHMP3, and CHMP6) and three regulatory subunits [34]. After cisplatin treatment, the ESCRT III subunits exhibited varying degrees of changes (Figure S3C-P). CHMP4 is an essential subunit in the ESCRT III complex, playing a crucial role in its function [35]. Therefore, in our study, we primarily focus on the overexpression of CHMP4 to exert the effects of ESCRT III and promote lysosomal repair. We transfected BUMPT cells with a plasmid encoding the major ESCRT III subunit CHMP4A tagged with GFP before being treated with cisplatin and our results show that the colocalization of LGALS3 and LAMP1 was reduced after CHMP4A overexpression (Figure 6A–D), which indicated that ESCRT III can repair lysosomal damage induced by cisplatin.

Then, we determine the influence of ESCRT III on autophagy in BUMPT cells. CHMP4A is a homologous protein to mouse CHMP4B with a similarity of 81% (Figure S4A). Following cisplatin treatment in BUMPT cells, the group with CHMP4B knockdown exhibited more severe autophagic dysfunction (Figure S4B-E). Immunofluorescence showed CHMP4A overexpression can effectively reduce the number of LC3 puncta and the expression of SQSTM1 and NBR1 induced by cisplatin (Figure 6E–J). Western blot showed the expression of NBR1, SQSTM1, CTSB, LC3-II, and GABARAPL1 induced by cisplatin were partly abolished by CHMP4A overexpression (Figure 6K–P). These results above confirm that ESCRT III can repair the autophagic disorders induced by cisplatin.

### ESCRT III protects against cisplatin-induced lysosomal damages and apoptosis in proximal tubular epithelial cells

**Figure 7.**
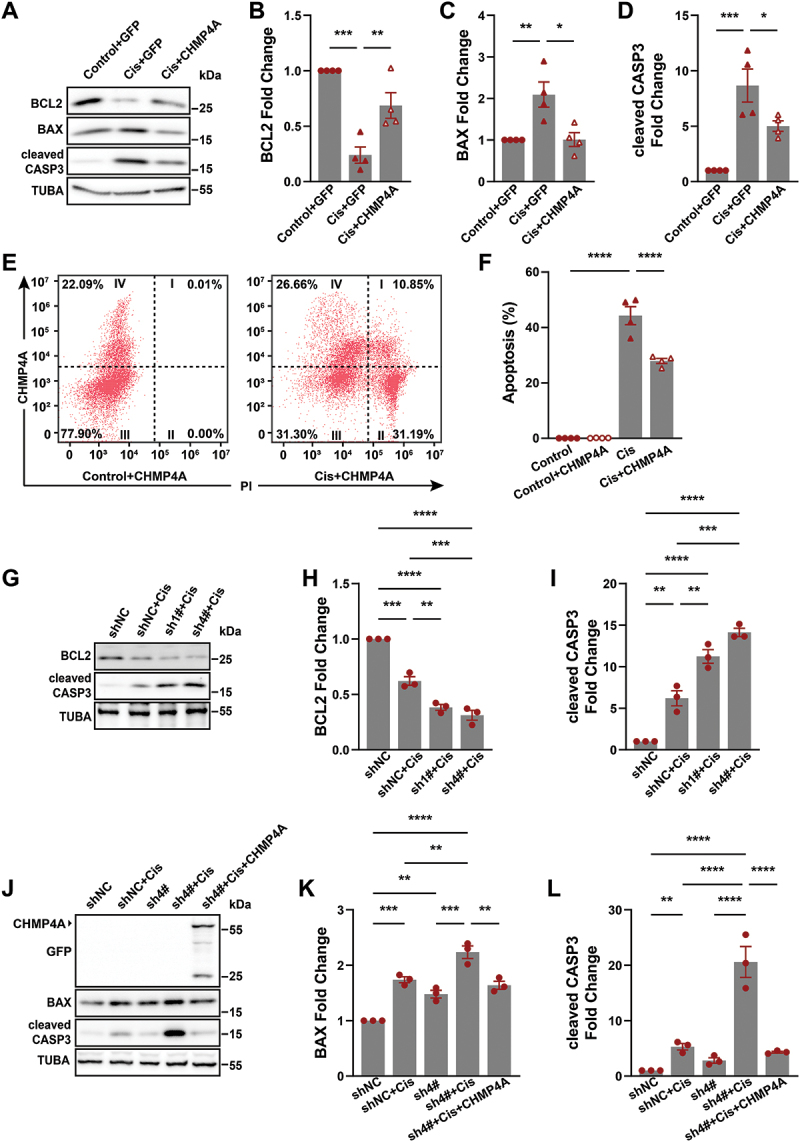
ESCRT III protects against cisplatin-induced apoptosis in BUMPT cells. (A) Western blot analysis of BCL2, BAX and cleaved CASP3 of BUMPT cells in each group as indicated. TUBA was used as the loading control. (B-D) Quantitative analysis of the data in (A) by ImageJ software. *p < 0.05, **p < 0.01, ***p < 0.001. (E) Flow cytometry analysis of PI staining of BUMPT cells in each group as indicated. (F) Percentages of apoptosis in (E) were quantified. Control+CHMP4A group in (E) was divided into control (II:[II+III]) and Control+CHMP4A (I:[I+IV]) groups. Cis+CHMP4A group in (E) was divided into cis (II:[II+III]) and Cis+CHMP4A (I:[I+IV]) groups. ****p < 0.0001. (G) Western blot analysis of BCL2 and cleaved CASP3 of BUMPT cells transfected with NC shRNA or CHMP4B shRNA, and then treated with DMSO or cisplatin. TUBA was used as the loading control. (H-I) Quantitative analysis of data shown in (G) using ImageJ software. **p < 0.01, ***p < 0.001, ****p < 0.0001. (J) Western blot analysis of GFP, BAX and cleaved CASP3 of BUMPT cells in each group as indicated. TUBA was used as the loading control. (K-L) Quantitative analysis of the data in (K) by ImageJ software. **p < 0.01, ***p < 0.001, ****p < 0.0001.

As ESCRT III ameliorate lysosomal damage and decrease expression of CTSB induced by cisplatin (Figure 6), the role of ESCRT III in cisplatin-induced apoptosis was studied. We first observed the effect of ESCRT III on nephrotoxicity of cisplatin in vitro. As showed in Figure 7A–D, the increased expression of cleaved CASP3, BAX and the decreased expression of BCL2 induced by cisplatin treatment were reversed by CHMP4A overexpression. Flow cytometry also showed that CHMP4A can inhibited cisplatin induced apoptosis in BUMPT cells (Figure 7E,F). The BCL2 expression decreased and cleaved CASP3 expression increased in BUMPT cells with CHMP4B knockdown indicating more severe cisplatin-treated apoptosis (Figure 7I,J). To further investigate, we performed overexpression experiments after CHMP4B knockdown. The results showed that CHMP4A overexpression reduced BAX and cleaved CASP3 expression levels (Figure 7J,K), suggesting that CHMP4A overexpression can alleviate cisplatin-induced apoptosis.

Additionally, it is worth mentioning that CHMP4 function also relies on ESCRT nucleating factors, including TSG101 and PDCD6IP [36]. Study found that CHMP4B requires the cooperative action of TSG101 and PDCD6IP to perform its membrane repair function [36]. Therefore, we performed a combined knockdown of Tsg101 and Pdcd6ip. First, we selected four siRNAs for each gene. After validation, si1–3# of *Pdcd6ip* showed the highest knockdown efficiency at 76.77%, and si2–1# of *Tsg101* showed the highest knockdown efficiency at 70.16% (Figure S5A and B). We then co-transfected si1–3# and si2–1# into cells to further verify the combined knockdown efficiency (Figure S5C and D). Our results showed that CHMP4A rescued SQSTM1, and cleaved CASP3 and BCL2 were observed without intervention, but after co-transfection of the *Tsg101* and *Pdcd6ip* siRNA, the CHMP4A rescue effect disappeared (Figure S6E-I). This result also indicates that the lysosomal repair function of CHMP4A requires the coordinated action of TSG101 and PDCD6IP.

**Figure 8.**
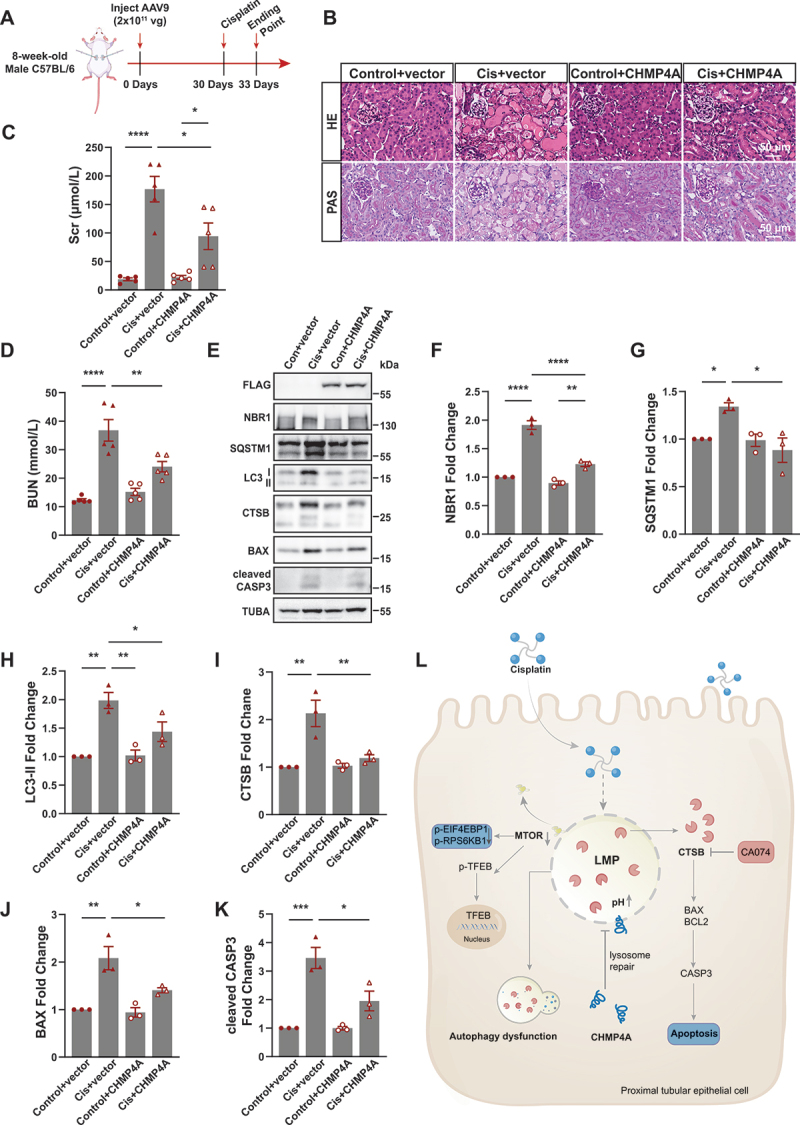
ESCRT III protects against cisplatin-induced lysosomal repair and apoptosis in proximal tubular epithelial cells of mice. (A) Flow diagram of mice with in situ injected with aav-vector or AAV-CHMP4A. (B) HE and PAS staining of kidney sections of mice with in situ injected with control-vector or control-CHMP4A, then treated with normal saline or cisplatin (25 mg/kg body weight). Scale bar: 50 μm. (C) Serum creatinine of mice in different treatment groups as indicated. *p < 0.05, ****p < 0.0001. *n* = 5. (D) BUN of mice in different treatment groups as indicated. **p < 0.01, ****p < 0.0001. *n* = 5. (E) Western blot analysis of flag, NBR1, SQSTM1, LC3, CTSB, BAX and cleaved CASP3 in the renal cortex of mice from each group as indicated. TUBA was used as the loading control. (F-K) Quantitative analysis of the data in (K) by ImageJ software. *p < 0.05, **p < 0.01, ***p < 0.001, ****p < 0.0001. *n* = 5. (L) A schematic summary of ESCRT iii-mediated lysosomal repair in cisplatin-induced renal tubular cell injury.

To determine the therapeutic potential of ESCRT III in AKI mouse model, we overexpressed ESCRT III in kidney of mice using an adeno-associated virus (AAV-vector) or AAV-CHMP4A by in situ injecting (Figure 8A). The efficiency of transfection was verified by immunofluorescence staining, as indicated in Figure S6. We subsequently focused on the effects of CHMP4A on cisplatin- induced renal tubule injury and cell apoptosis. Results showed CHMP4A overexpression improved renal damage confirmed by HE staining and PAS staining (Figure 8B) and decreased serum creatine and BUN concentrations (Figure 8C,D). These findings indicate that ESCRT III mitigates renal damage in cisplatin-induced AKI. As expected, western blot analysis showed that AAV-CHMP4A markedly decreased CTSB, NBR1, SQSTM1 and LC3-II by western blot (Figure 8E–I), suggesting an improvement in lysosomal function in cisplatin-induced AKI. Furthermore, AAV-CHMP4A significantly reduced cleaved CASP3 and BAX levels (Figure 8E,J,K), indicating that ESCRT III alleviates apoptosis in cisplatin-induced AKI.

## Discussion

The nephrotoxicity caused by cisplatin is one of the key reasons limiting its clinical application. Although many studies have focused on its pathogenesis, the incidence of cisplatin-induced AKI remains high and there are no clear approved therapeutics [37]. In this study, we investigate the role of the lysosomal damage and repair in the development of cisplatin-induced AKI. We found that cisplatin caused renal tubular cell apoptosis and autophagy disorder, which may due to lysosomal damage. Moreover, we discovered that the ESCRT III subunit CHMP4A can alleviate cisplatin-induced AKI by repairing lysosomal damage, thereby improving autophagy dysfunction and inhibiting cell apoptosis in renal tubular cells. Our study may provide valuable strategies to mitigate cisplatin-induced AKI.

The lysosome is a membrane-bound organelle with acidic pH, and is associated with cell death via LMP leading to lysosome-dependent cell death [38]. The distinctive sign of LMP is the translocation of soluble lysosomal components (including enzymes) from the lysosomal lumen to the cytosol [30]. In our study, LysoTracker and LysoSensor staining indicated cisplatin abolished lysosomal acidification in BUMPT cells. Meanwhile, cisplatin induced the translocation of CTSB from lysosomes to the cytosol. Given this, LMP was induced by cisplatin in PTECs. Depending on the cell type, the nature and intensity of the stimulus, LMP can initiate or amplify different types of cell death. There is evidence to suggest that CTSB translocated from lysosome into the cytosol is critical for NLRP3 inflammasome activation, which is a core component of the pyroptosis pathway [39]. Pretreatment with CA074 protected HT22 cells against glutamate and erastin induced ferroptosis [40]. In addition, LMP was involved in the execution of other types of cell death such as apoptosis, autophagy and necrosis [41]. To further validate whether cytosolic location of CTSB was involved in cisplatin-induced apoptosis, the specific cathepsin inhibitors, CA074 was pretreated with BUMPT cells block the activities of CTSB. Results showed cisplatin-induced CASP3 activation and consequent elevated apoptosis were significantly prevented by the inhibition of cathepsins. Collectively, these data indicate that LMP have an important role in cisplatin-induced apoptosis of PTEC cells.

Lysosomes are the main degradative organelles in the cell, play an important role in autophagy. The fusion of lysosomes with autophagosomes to form autolysosomes is critical for autophagic flux, while impairment of autophagosome – lysosome fusion will inhibit the degradation of autophagosomes. Numerous factors, including silica, drugs, urate crystals and oxidative stress, induce lysosomal membrane permeabilization and/or lysosomal rupture. Lysosome damage increased LC3 lipidation, and lipidated LC3 is localized on the autophagosome membrane. LC3 lipidation was essential for TFEB activation, which is critical to prevent crystal nephropathy [42]. Lysosomal damage suppresses MTOR activity through LGALS8 and activates AMPK through LGALS9. LGALS8 and LGALS9 were both contributed to protect against Mycobacterium tuberculosis [43]. Under conditions of lysosomal damage, loss of autophagy inhibited lysosomal biogenesis and deteriorated acute kidney injury [44]. In our study, the level of LC3-II was found to be increased upon cisplatin treatment, indicating an upregulation in LC3-II lipidation. Additionally, we observed an increase in autophagy receptors SQSTM1 and NBR1, suggesting that cisplatin induces autophagy dysfunction. Disruption of lysosomal integrity can activate signaling pathways that influence TFEB activity, forming a crucial feedback loop to enhance lysosomal enzyme production and activity, thereby supporting lysosomal repair and degradation processes [10]. This is consistent with our experimental results, cisplatin inhibited the phosphorylation of MTOR substrates EIF4EBP1 and RPS6KB1 while promoting the nuclear translocation of TFEB, providing further evidence for its inhibitory effect on MTOR activity and subsequent lysosomal damage TFEB, a key regulator of lysosomal biogenesis and autophagy, promotes the expression of genes related to lysosomal function, including CTSB [45]. When ESCRT III is overexpressed and lysosomal integrity is restored, the TFEB feedback loop is diminished, and CTSB levels decrease.

Lysosomes are essential organelles that carry out degradation and signaling functions. As there is a low intralumenal pH and high content of Ca^2+^ and enzymes in the lysosome, loss of lysosomal function due to LMP is highly deleterious and can induce inflammation and even cell death [13,46]. On the one hand, in response to LMP, ESCRT complexes were recruited rapidly to damaged lysosomes precedes LGALS3 and lysophagy. While interference with ESCRT recruitment caused cell death, indicating that ESCRT-mediated lysosomal repair plays an important role in promoting cell viability after lysosome injury [36,47]. In addition, LMP can be mitigated by lipid scrambling and phosphoinositide-regulated transport of lipids from the ER at ER-lysosome contact sites. Independently of ESCRT, lysosomal damage triggered a rapid calcium-activated sphingomyelin scrambling and turnover to mediate lysosomal repair [48]. LMP stimulates a PITT pathway for rapid lysosomal repair, which plays an important role in numerous age-related diseases characterized by impaired lysosomal function [49]. On the other hand, Lysosomes with severe damage which is too extensive to be repaired can be selectively degraded by macroautophagy, known as lysophagy [50]. In our study, ESCRT III subunit CHMP4A can alleviate cisplatin-induced AKI by repairing the lysosomal damage, further improved apoptosis and autophagy disorders. Whether other mechanism of lysosomal repair or lysophagy is involved in cisplatin induced AKI need to be further studied.

In conclusion, our results demonstrated ESCRT III mediated lysosomal repair play an important role in cisplatin-induced AKI, partly through improving cell apoptosis and autophagy disorder (Figure 8L).

## Materials and methods

### Reagents, antibodies and plasmids

DMEM was obtained from Gibco (C11995500BT). Fetal bovine serum was obtained from Sigma-Aldrich (f8318). LLOMe was purchased from Sigma-Aldrich (L7393). Torin1 (S2827) and CQ (S6999) were obtained from Selleck. CA074 was sourced from MedChemExpress (HY-103350). Lipofectamine 2000 was obtained from Thermo Fisher Scientific (11668019). The following antibodies were used in the experiments: anti-LC3B (Cell Signaling Technology, 2775, 12741), anti-GABARAPL1 (Cell Signaling Technology 13,733) anti-SQSTM1/p62 (Sigma-Aldrich, P0067), anti-LAMP1 (Santa Cruz Biotechnology, sc-18,821, sc-20,011), anti-CTSB/cathepsin B (Cell Signaling Technology 31,718), anti-NBR1 (Cell Signaling Technology 20,145), anti-BAX (proteintech 50,599–2-Ig), anti-BCL2 (Cell Signaling Technology, 3498), anti-cleaved CASP3/caspase 3 (Cell Signaling Technology, 9661), anti-EIF4EBP1 (Cell Signaling Technology, 9644), anti-p-EIF4EBP1 (Cell Signaling Technology, 2855), anti-RPS6KB1 (Cell Signaling Technology 34,475), anti-p-RPS6KB1(Cell Signaling Technology, 9209), anti-LGALS3/galectin 3 (Cell Signaling Technology 89,572), anti-TFEB (Cell Signaling Technology 83,010), anti-p-TFEB (Cell Signaling Technology 37,681), anti-GFP (Cell Signaling Technology, 2956), anti-Flag (Cell Signaling Technology, 14793S), anti-CHMP2A (proteintech 10,477–1-AP), anti-CHMP2B (proteintech 12,527–1-AP), anti-CHMP4B (proteintech 13,683–1-AP), anti-CHMP6 (proteintech 16,278–1-AP), anti-AQP1/aquaporin 1(abcam, ab168387). Peroxidase AffiniPure Goat Anti-Mouse (115–545–174), Peroxidase AffiniPure Goat Anti-Rat (112-545-062, 112-605-062), and Peroxidase AffiniPure Goat Anti-Rabbit (111-545-144, 111-165-003) were from Jackson ImmunoResearch Laboratories. Plasmids GFP-TFEB, GFP-CHMP4A, and TF-LC3 were confirmed by sequencing.

### Mice and treatment

Male C57BL/6 mice, weighing 20–25 g and aged 8–10 weeks, were obtained from BORESEN BIOTECH (Hunan, China) and randomly assigned to either the control group or the cisplatin group, with 8 mice per group. Cisplatin (10 mg; Qilu Pharmaceutical, H20023460), obtained from the Department of Oncology, Third Xiangya Hospital of Central South University, was diluted to a concentration of 2 mg/ml using normal saline. The cisplatin group received intraperitoneal injection of cisplatin at a dosage of 25 mg/kg body weight, while the control group received an equivalent volume of normal saline via intraperitoneal injection. All animals were euthanized at 72 h after cisplatin injection, and blood samples and renal tissues were collected for further analysis. All animal experiments were approved by the Laboratory Animal Ethics Review Committee of Central South University (Approval no. XMSB-2023–0358).

### Serum biochemical index analysis

Weights were recorded at the beginning and end of the experiment, with daily monitoring of weight changes. Serum creatinine (Scr) and blood urea nitrogen (BUN) levels were analyzed using an automated biochemical analyzer (Perkinelmer EnSight, Singapore) at The Third Xiangya Hospital, Central South University.

### Renal histology

Kidney tissues were fixed in 4% neutral buffered formalin, dehydrated using graded alcohol, and embedded in paraffin. Sections of 4-μm thickness were then prepared and stained with PAS and HE for histological examination.

### Immunofluorescence staining

Changes in lysosomal damage (LAMP1, LGALS3), autophagy (LC3-II, SQSTM1, NBR1), and apoptosis (cleaved CASP3) were assessed via immunofluorescence staining of frozen tissues. Tissues were sectioned into 30-μm thickness and placed in 24-well plates containing 1×PBS containing 137 mM NaCl (Sigma-Aldrich, S5886), 10.1 mM Na_2_HPO_4_ (Sigma-Aldrich, S5136), 2.68 mM KCl (Sigma-Aldrich, P5405), 1.76 mM KH_2_PO_4_ (Sigma-Aldrich, P5655), pH 7.2 for bleaching, while cells were fixed with 4% paraformaldehyde in PBS for 10 min at room temperature (RT). Following permeabilization with PBST (PBS with 0.1% Triton X-100 [Sigma-Aldrich, T8787]) for 15 min, samples were blocked with BSA (Abiowell, AWB0157e) for 1 h at RT, followed by overnight incubation at 4°C with primary antibodies (1:200). After PBS washing, sections were incubated with Alexa Fluor-conjugated goat anti-rabbit secondary antibody (Jackson, 111-035-144) for 1 h at RT, washed again with PBS, and counterstained with DAPI to visualize the nuclei. Confocal imaging was performed using a LSM 900 inverted confocal microscope. Experiments were repeated more than three times. Image files were viewed and analyzed using ImageJ software.

### TUNEL assay

TUNEL assay was conducted using the TUNEL BrightRed Apoptosis Detection Kit (Vazyme, A113) following the manufacturer’s instructions. After permeabilizing the kidney tissue sections for 20 min, incubate them with the TUNEL reagent in the dark at 37°C for 1 h, followed by staining with DAPI. Apoptotic renal cells exhibit red fluorescence. Images of multiple kidney tissues from three independent experiments were captured using a microscope.

### Transmission electron microscopy

In brief, 1-mm^3^ sample of fresh renal cortex was promptly transferred to a 4°C electron microscope incubator and sent to the TEM laboratory at Xiangya Hospital, Changsha, Hunan, China. The tissue was embedded, sliced into 60–80 nm ultrathin sections, and double-stained with uranium and lead. Subsequent transmission electron microscopy allowed for the observation of lysosomal changes in renal tubular epithelial cells and image acquisition.

### In situ injection of adeno-associated virus to kidneys

AAV2/9 vectors carrying CHMP4A overexpression sequences (pcAAV-CMV-CHMP4A-GdGreen-3×FLAG) or the negative control (pcAAV-CMV-GdGreen) were produced by OBio Technology (Shanghai, China). The vectors were delivered to the renal cortex using a 10-μl microsyringe for multipoint injections. Briefly, the C57 mice were anesthetized with isoflurane and maintained on a heating pad to preserve body temperature. Two small incisions (approximately 1 cm each) were made in the back to expose both kidneys. Carefully puncture the renal cortex and slowly inject 8 μl of the carrier at each site. After injection and needle withdrawal, apply a dry cotton swab for 1 min to control hemostasis and prevent fluid leakage. Following the manufacturer’s instructions, adeno-associated virus (AAV) was injected at a volume of 80 μl (approximately 2 × 10^11^ vg) per mouse. The ten injection sites were distributed across the middle of both kidney sides, the upper and lower poles, and the middle of the outer edge. Eight-week-old C57BL/6 mice were injected with AAV virus and monitored for 30 days, followed by cisplatin treatment. Blood and tissue samples were then collected three days later.

### Cell culture and treatment

The Boston University mouse proximal tubular cell line (BUMPT) was provided by the Institute of Kidney Disease, Central South University. Cisplatin was acquired from Beyotime (ST1164). BUMPT cells were exposed to varying concentrations of cisplatin (0, 1.25, 2.5, 5, 10 and 20 μM), and alterations in cell damage were visualized under the microscope (Motic, AE31E). To evaluate cisplatin-induced lysosomal damage, BUMPT cells were treated with cisplatin (5 μM, 24 h), with LLOMe (1 mM, 3 h) as a positive control. To assess the effects of cisplatin on autophagy and apoptosis, cells were exposed to cisplatin (0, 1.25 and 5 μM) for up to 24 h. To explore the mechanism of lysosomal damage, BUMPT cells were exposed to cisplatin (5 μM), with torin1 (1 μM, 1 h) as a positive control. To investigate the localization changes of CTSB after cisplatin treatment, the cells were pre-incubated with CA074 (100 μM, 3h) during the 24-hour cisplatin (5 μM) treatment. To evaluate lysosomal, autophagic, and apoptotic repair, changes were observed following transfection with GFP-CHMP4A plasmid.

### Western blot analysis

Kidney cortex or BUMPT cells total protein was quantified using a BCA protein assay kit (Thermo Fisher Scientific 23,225). Samples were separated via SDS/PAGE and transferred to PVDF membranes (Millipore, IPVH00010). Membranes were blocked with PBST containing 5% nonfat milk for 1 h, followed by overnight incubation at 4°C with primary antibodies. After three PBST washes, membranes were incubated with HRP-conjugated goat anti-mouse (Jackson, 115-005-003) or anti-rabbit IgG antibodies (Jackson, 111-035-144) at room temperature for 1 h. Protein expression levels were detected using chemiluminescent staining reagent kits (SuperSignal West Femto 34,095), and images were captured with an Image Scanner. Experiments were repeated more than three times. Band intensities were quantified using Fiji software.

### Subcellular fractionation and in vitro fusion assay

Subcellular fractionation was performed with minor modifications [51]. BUMPT cells were divided into three groups: control, cisplatin (5 μM for 24 h), and BafA1 (100 nM for 2 h), with each group containing four 10-cm dishes. After digestion, cells were collected by centrifugation at 1000 × g for 10 min, washed with pre-chilled PBS, and resuspended in 7–8 mL homogenization buffer (250 mM sucrose [Sigma-Aldrich, V900116], 10 mM HEPES [Sigma-Aldrich, H3375], pH 7.4, 1 mM EDTA [Sigma-Aldrich, E9884], and a protease inhibitor cocktail [Sigma-Aldrich 11,836,170,001] [52]). The suspensions were homogenized using a Dounce homogenizer (Kimble 885,300–0007) with a type B pestle for 15–20 strokes. Lysates were then centrifuged at 1,000 × g for 10 min at 4°C to remove debris. The floating fat layer was discarded, and the supernatant was retained. The resulting pellet was resuspended in two volumes of homogenization buffer, gently homogenized for an additional 3–4 strokes, and centrifuged again. All supernatants were pooled and subjected to further centrifugation at 70,000 × g for 20 min at 4°C to isolate the cytosolic fraction. The pellet was resuspended in 0.87 mL homogenization buffer and mixed with 1.45 mL of 80% (wt:vol) Histodenz (Sigma-Aldrich, D2158) stock solution (80% Histodenz, 1 mM EDTA, and 10 mM HEPES, pH 7.4). A discontinuous Histodenz gradient (15, 20, 24 and 26%) containing protease inhibitors was layered on top of the sample. The mixture was centrifuged at 110,000 × g for 4 h at 4°C. Fractions containing autophagosomes (AP) and lysosomes (LY) at the gradient interfaces were collected using a syringe. Collected fractions were diluted to 0.01 µg/µL protein in fusion buffer (10 mM HEPES, pH 7.0, 10 mM KCl, 1.5 mM MgCl_2_, 1 mM DTT [MedChemExpress, HY-15917], 250 mM sucrose, and protease inhibitor cocktail). AP were labeled with an anti-LC3 antibody followed by a CY3-conjugated secondary antibody (Jackson ImmunoResearch, 111-165-003), while LY were labeled with an anti-LAMP1 antibody and an Alexa Fluor 488–conjugated secondary antibody (Jackson, 112-545-062). The fusion assay was conducted in an energy-regenerating system containing 3 mM ATP (MedChemExpress, HY-B2176), 2 mM GTP (ThermoFisher, R0461), 2 mM CaCl_2_, 8 mM phosphocreatine (MedChemExpress, HY-D0885), 0.16 mg/mL creatine phosphokinase (MedChemExpress, HY-P2799), and a protease inhibitor cocktail, incubating at 37°C for 40 min. To evaluate the effects of BafA1(Selleck, S1413), vesicles were pretreated with the compound for 20 min before the fusion reaction. The reaction was terminated by adding 8% paraformaldehyde (PFA) on ice for 15 min. Samples were mounted on slides using 50% glycerol (Sigma-Aldrich, G2025) and visualized with a Leica TCS SP8 confocal microscope. Experiments were repeated more than three times. Fusion events were quantified with ImageJ and expressed as the percentage of total vesicles.

### shRNA and siRNA silencing

shRNAs and siRNAs were purchased from GenePharma (Shanghai, China). The shRNA sequences used were targeting mouse *Chmp4b*: sh1# sequence (5′ GGG ACA CGG AGG AGA TGT TAA) and sh4# sequence (5′ GGC AGA GTT GGA GGA ACT TGA), or firefly luciferase (5′ GTT CTC CGA ACG TGT CAC GT) as a nontargeting control. The siRNA sequences used were against mouse *Tsg101*: si2–1# sequence (5′ GCG UUA UCG AGG UAA UAU ATT), *Pdcd6ip*: si1–3# sequence (5′ CCA UCG CAC UUC UGU GUA ATT), or (5′ UUC UCC GAA CGU GUC ACG UTT) as a nontargeting control. BUMPT cells were transfected with the indicated shRNAs and siRNAs using the Lipofectamine 2000 reagent according to the manufacturer’s recommendations.

### Real-time PCR

Total RNA isolated using Trizol reagent (Invitrogen 15,596–018) was converted to cDNA with the HiScript II Q RT SuperMix for qPCR (+gDNA wiper) (Vazyme, R223–01) according to the manufacturer’s instruction. SYBR qPCR Master Mix (Vazyme, Q711–02) was used for quantitative real-time PCR amplification with a Real-Time PCR Detection System (Quantagene q225MX–400) and corresponding software. Primers for *Chmp4b* were 5′- GGGACACGGAGGAGATGTTA-3′ (forward) and 5′- ACCTCTTCTTGCGCTTCAGA-3′ (reverse). Primers for *Chmp3* were 5′- AGGAGAAGCCTCCCAAAGAG-3′ (forward) and 5′- TGGCTGCATCTTTCACAGAC-3′ (reverse). Primers for *Chmp2a* were 5′- AACTGGACAGGGAACGACAG-3′ (forward) and 5′- CCAGGTCTTTTGCCATGATT-3′ (reverse). Primers for *Chmp2b* were 5′- GACCGAGCAGCCTTAGAGAA-3′ (forward) and 5′- GTTTCCGTAGGTGGACAAGC-3′ (reverse). Primers for *Chmp6* were 5′- GAGTCACCGAACAGGACAGG-3′ (forward) and 5′- CTCGTTCTTTCCTGCCATCC-3′ (reverse). Primers for *Tsg101* were 5′- CGTCCGTCAAACTGTCAATG-3′ (forward) and 5′- CTCGATAACGCACTGGGATT-3′ (reverse). Primers for *Pdcd6ip* were 5′- CGAGGAGCTCAGCAAACTG-3′ (forward) and 5′- ATGGGAACTTGGGTTCAATG-3′ (reverse). Primers for *Sqstm1* were 5′- CGAGTGGCTGTGCTGTTC-3′ (forward) and 5′-TGTCAGCTCCTCATCACTGG-3′ (reverse). Primers for *Nbr1* were 5′-CCAGATAAAATACCTGGATGAGG-3′ (forward) and 5′-CATGGTACCCTTCGTGGACT-3′ (reverse). PCR was performed with 1 cycle at 94°C for 30 s, followed by 45 cycles at 95°C, 15 s, and 60°C, 30 s. Gene expression was normalized to actin, and relative mRNA levels were calculated based on the comparative Ct method. Experiments were repeated more than three times.

### LysoTracker and LysoSenser staining

LysoTracker (L7528) and LysoSensor (L7545) were obtained from Thermo Fisher Scientific. After HBSS (Gibco 24,020–117) washing, BUMPT cells were incubated with the LysoTracker (75 nM) in the dark at 37°C for 1 h, followed by staining with LysoSenser (1 μM) at 37°C for 1 h. Images of multiple cells from three independent experiments were captured using a microscope.

### PI staining

The Annexin V/PI Apoptosis Detection Kit (KTA0002) was obtained from Abbkine. Transfected BUMPT cells were exposed to 0, 1.25 and 5 μM cisplatin for 24 h to evaluate cisplatin-induced apoptosis. BUMPT cells were stained with 2 μL of propidium iodide (PI) at room temperature for 15 min in the dark, followed by Hoechst 33,342 (Beyotim, C1022) staining for 15 min. Images of multiple cells were acquired from three independent experiments using a microscope.

### CCK-8 assay

The BUMPT cells viability was detected by CCK-8 Cell Counting Kit (Vazyme, A311) following the manufacturer’s instruction. Briefly, BUMPT cells were seeded into 96-well plates at a density of 5 × 10^3^ cells per well. After treatment with different reagents, the culture medium in each well was replaced with 100 μL CCK-8 detection solution (90 μL fresh medium mixed with 10 μL CCK-8 solution). The time of color reaction with CCK-8 in each test was fixed to 2 h to avoid irrelevant variables. Optical density (OD) values were detected with a spectrophotometer (Perkin Elmer EnSight) at 450 nm. Experiments were repeated more than three times.

### Flow cytometry

The transfected BUMPT cells were exposed to cisplatin for 24 h to assess the lysosomal repair following cisplatin-induced apoptosis. After digestion with EDTA-free trypsin, cells were collected and suspended in 100 μL of binding buffer. Subsequently, BUMPT cells were stained with 2 μL of PI at room temperature in the dark for 15 min. Finally, 400 μL of binding buffer was added before analyzing the cell distribution using flow cytometry. Experiments were repeated more than three times.

### Statistical analysis

Data analyzed via Prime 10.1.1 software, presented as mean ± standard error of the mean (SEM). Qualitative data represent ≥ 3 experiments. Statistical differences in multiple groups determined by ANOVA followed by Tukey’s post-hoc tests. Student’s t-test used for between-group differences. Immunofluorescence colocalization statistical analysis was performed using Manders’ correlation analysis. *p* < 0.05 denotes statistical significance.

## Authors’ contributions

Zhangyu Tian and Yiming Wu contribute equally to this work. Zhangyu Tian and Yiming Wu contributed to the testing, data analysis and interpretation. Zhangyu Tian and Aimei Li wrote the first draft of the manuscript. Hao Zhang and Aimei Li contributed to the study design and manuscript revision. Bin Yi and Ling Li were involved in Serum biochemical index analysis and the induction of the AKI model. Yan Liu completed some cellular experiments during the revision process. All authors read and approved the final manuscript.

## Supplementary Material

Supplementary materials R3.docx

## Data Availability

All data generated or analyzed during this study are included in this published article.
