# Supplementary material for: ESCRT III-mediated lysosomal repair improve renal tubular cell injury in cisplatin-induced AKI
Source: Autophagy. 2025 Apr 4;21(9):1927–44. doi: 10.1080/15548627.2025.2483598 (PMC12366826; doi:10.1080/15548627.2025.2483598)
Supplement: Supplementary materials R3.docx [file KAUP_A_2483598_SM1542.docx]

**Supplementary Figure legends**

**
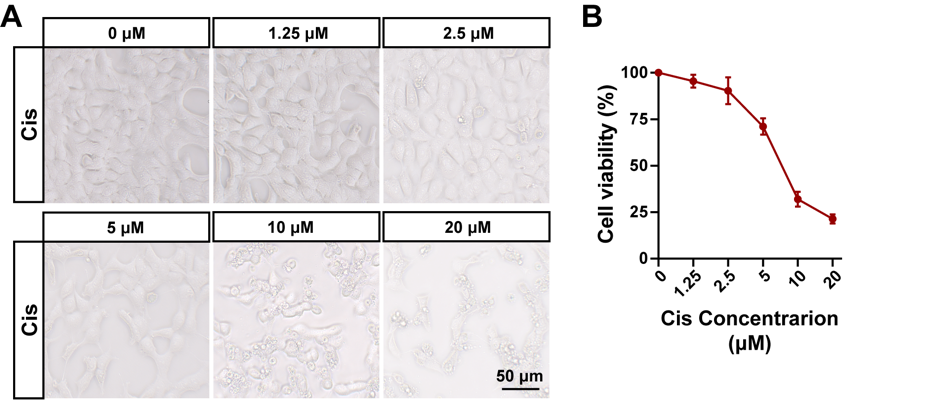
**

**Figure S1.** Cell viability of different concentrations of cisplatin on BUMPT cells. (**A**) Cell morphologies after intervention of cisplatin with different. Scale bar: 50 μm. (**B**) CCK-8 assay for BUMPT cells viability after intervention of cisplatin with different concentrations. Data are expressed as the mean ± standard error of the mean (SEM).


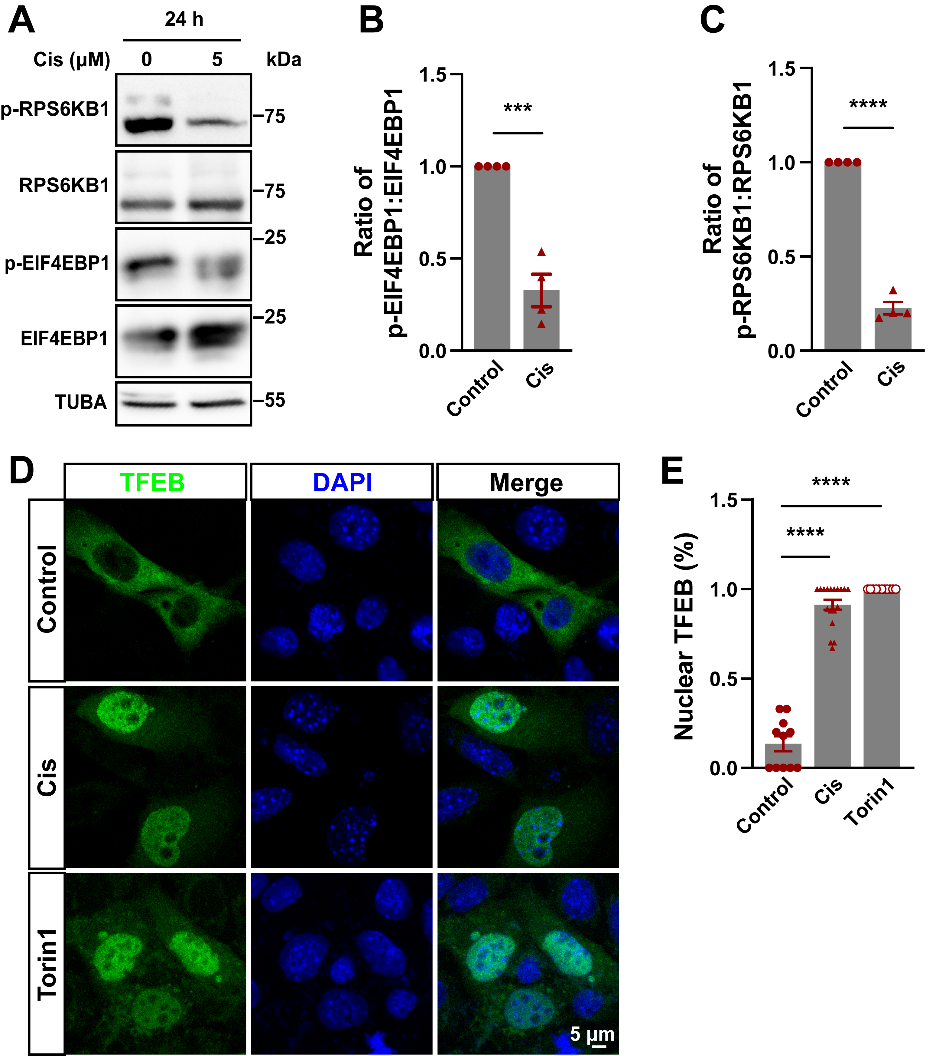


**Figure S2.** Cisplatin inhibited MTOR activity in BUMPT cells. (**A**) Western blot analysis of p-EIF4EBP1, t-IEF4EBP1, p-RPS6KB1 and t-RPS6KB1 in BUMPT cells of each group as indicated. TUBA/α-tubulin was used as the loading control. (**B-C**) Quantitative analysis of the data in (A) by ImageJ software. *p < 0.05, ***p < 0.001 vs Control group. (**D**) Immunofluorescence analysis of TFEB after transfection with GFP-TFEB in BUMPT cells, and then treatment with DMSO, cisplatin or Torin1. Scale bar: 5 μm. (**E**) Percentages of nuclear TFEB in (D) were quantified. ****p < 0.0001 vs Control group.

**
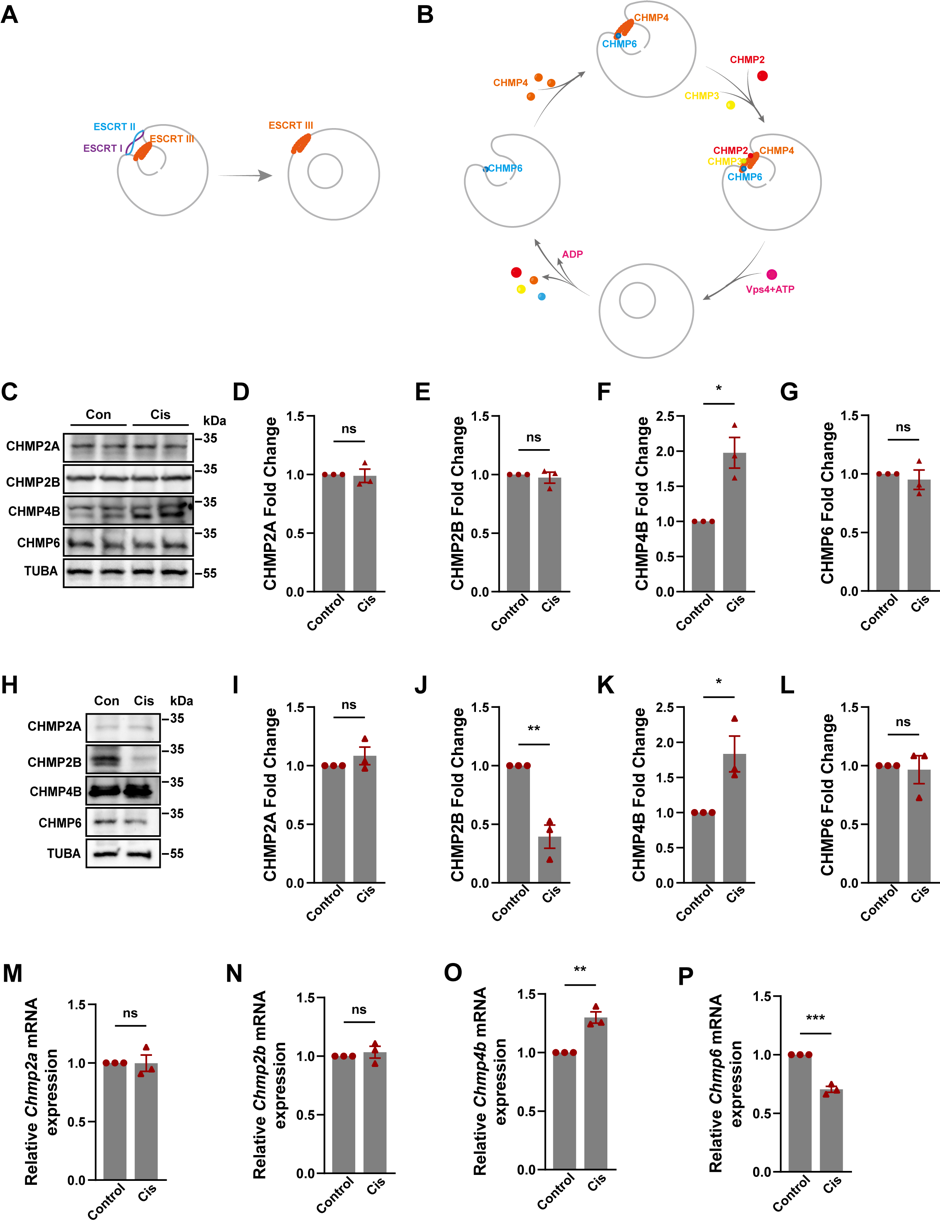
**

**Figure S3.** Effects of cisplatin on the ESCRT III subunits. (**A**) The role of the ESCRT family in membrane repair. (**B**) The dynamic repair process of the ESCRT III subunits. (**C**) Western blot analysis of CHMP2A, CHMP2B, CHMP4B and CHMP6 in the renal cortex of mice from each group as indicated. TUBA was used as the loading control. (D-G) Quantitative analysis of the data in (C) by ImageJ software. ns: no significant. *p < 0.05 vs Control group. n = 8. (H) Western blot analysis of CHMP2A, CHMP2B, CHMP4B and CHMP6 of BUMPT cells in each group as indicated. TUBA was used as the loading control. (I-L) Quantitative analysis of the data in (C) by ImageJ software. ns: no significant. ns: no significant. *p < 0.05, **p < 0.01vs Control group. (M-P) Changes of mRNA expression in genes encoding ESCRT III subunits in BUMPT cells as observed by qPCR. ns: no significant, **p < 0.01, ***p < 0.001 vs Control group.

**
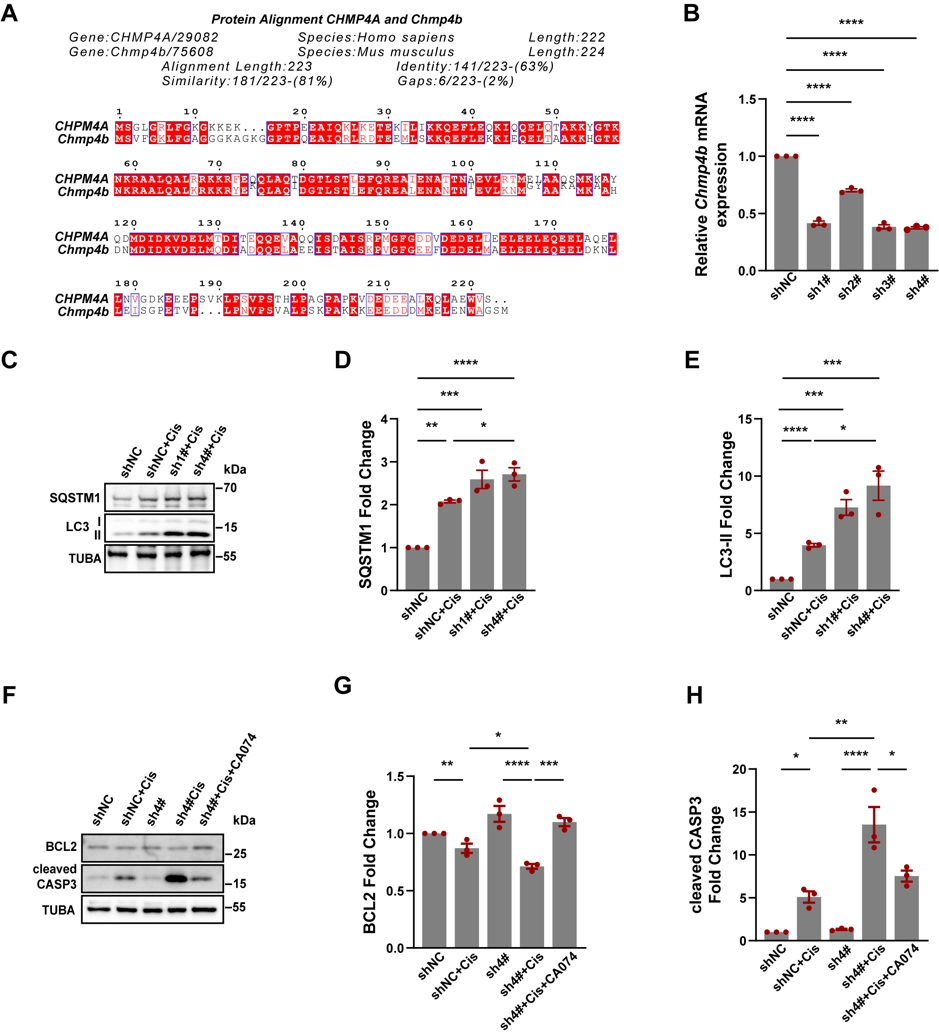
**

**Figure S4.** Knockdown of Chmp4b exacerbates cisplatin-induced autophagy. (**A**) Alignment of human *CHMP4A* and mouse *Chmp4b* sequences. (B) qPCR was used to evaluate the knockout efficiency of four *Chmp4b*-targeting shRNAs. ****p < 0.0001 vs shNC group. (C) Western blot analysis of SQSTM1 and LC3 of BUMPT cells in each group as indicated. TUBA was used as the loading control. (D-E) Quantitative analysis of data shown in (C) using ImageJ software. *p < 0.05, **p < 0.01, ***p < 0.001, ****p < 0.0001. (F) Western blot analysis of BCL2 and cleaved CASP3 of BUMPT cells in each group as indicated. TUBA was used as the loading control. (G-H) Quantitative analysis of data shown in (F) using ImageJ software. *p < 0.05, **p < 0.01, ***p < 0.001, ****p < 0.0001.

**
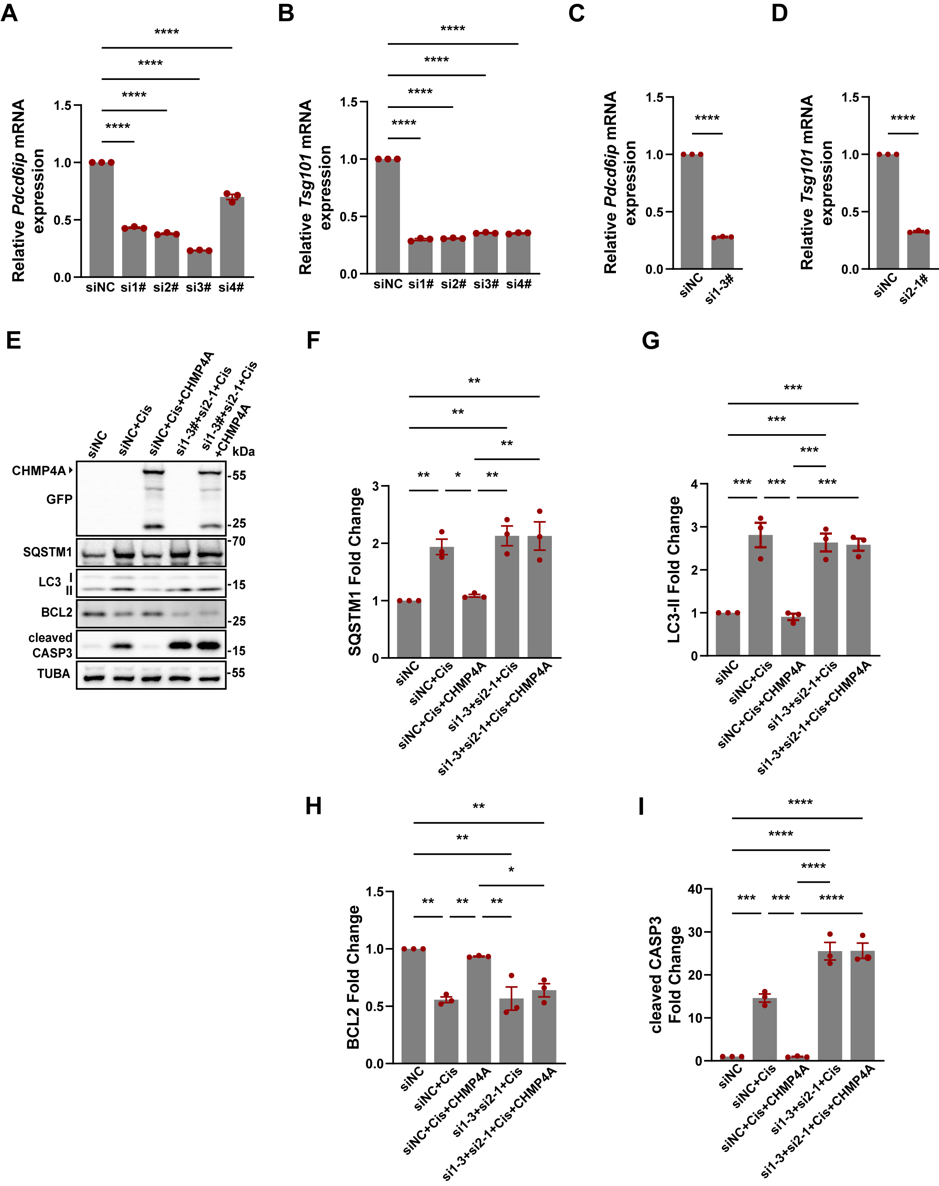
**

**Figure S5.** CHMP4 function relies on ESCRT nucleating factors TSG101 and PDCD6IP. (**A**) qPCR was used to evaluate the knockout efficiency of four *Pdcd6ip*-targeting siRNAs. ****p < 0.0001 vs siNC group. (B) qPCR was used to evaluate the knockout efficiency of four *Tsg101*-targeting siRNAs. ****p < 0.0001 vs siNC group. (C-D) qPCR was used to assess the knockout efficiency after co-transfection with siRNAs targeting *Pdcd6ip* and *Tsg101*. ****p < 0.0001 vs siNC group. (E) Western blot analysis of GFP, SQSTM1, LC3, BCL2 and cleaved CASP3 of BUMPT cells in each group as indicated. TUBA was used as the loading control. (F-I) Quantitative analysis of data shown in (E) using ImageJ software. *p < 0.05, **p < 0.01, ***p < 0.001, ****p < 0.0001.


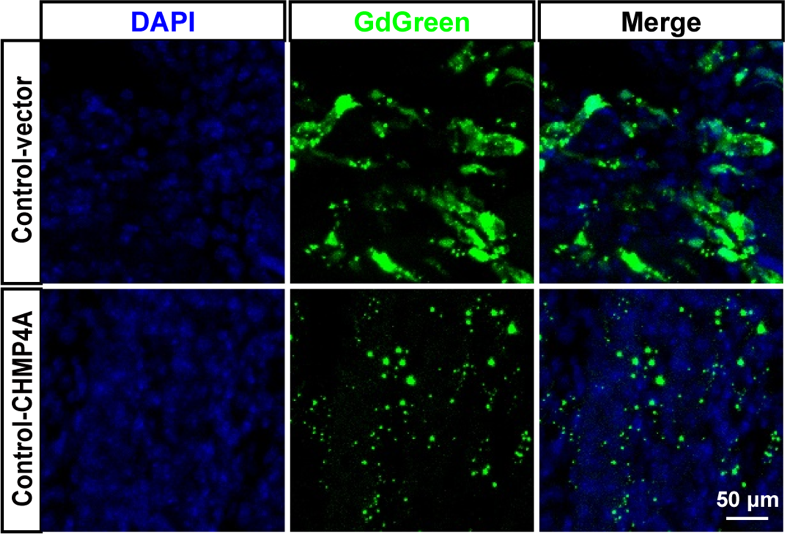


**Figure S6.** Expression of AAV in kidney. Immunofluorescence analysis of GdGreen in kidney section of mice with in situ injection of adeno-associated virus (Control-vector or Control-CHMP4A). Scale bar: 50 μm.
